# Effect of Novel Remodeled Bicycle Pedal Training on Balance Performance in Athletes With Functional Ankle Instability

**DOI:** 10.3389/fbioe.2020.600187

**Published:** 2020-10-22

**Authors:** Yi-Shuo Chang, Md Samsul Arefin, Yu-Lin You, Li-Chieh Kuo, Fong-Chin Su, Hong-Wen Wu, Cheng-Feng Lin

**Affiliations:** ^1^Department of Physical Therapy, College of Medicine, National Cheng Kung University, Tainan, Taiwan; ^2^Department of Biomedical Engineering, College of Engineering, National Cheng Kung University, Tainan, Taiwan; ^3^Department of Occupational Therapy, College of Medicine, National Cheng Kung University, Tainan, Taiwan; ^4^Department of Physical Education, National Taiwan University of Sport, Taichung, Taiwan; ^5^Physical Therapy Center, National Cheng Kung University Hospital, Tainan, Taiwan

**Keywords:** ankle sprain, remodeled bicycle pedal training, single-leg standing, proprioception, postural balance

## Abstract

**Context:**

Appropriate training without risk of injury is a critical concern for athletes. Remodeled bicycle pedal training with multi-directional challenges may be effective in improving the balance performance of athletes with functional ankle instability (FAI).

**Objective:**

To evaluate the effects of 6-week modified bicycle pedal training on the balance ability and proprioception of athletes with FAI.

**Design:**

Randomized controlled trial.

**Setting:**

University motion analysis laboratory.

**Participants:**

Fourteen healthy athletes (healthy group) and twenty-six athletes with FAI and an age of 18 to 30 years old. The participants with FAI were randomly distributed to two groups, designated as the training group (AI-T group) and non-training group (AI-NT group), respectively. The athletes in the AI-T group received 6-week remodeled bicycle pedal training, while those in the AI-NT group received no intervention at all.

**Intervention:**

A 6-week training using modified bicycle pedal capable of moving freely during loading cycle vs no intervention.

**Main Outcome Measures:**

The passive ankle joint position sense (JPS) in four angles and the center of pressure (COP) parameters were analyzed during single-leg standing with and without vision, respectively.

**Results:**

A 6-week remodeled pedal training: (1) significantly improved the passive JPS of ankle in all directions (*P* < 0.05); (2) reduced the excursion of the COP in the medial-lateral (ML) direction (*p* < 0.05), the velocity of the COP in the ML direction (*p* < 0.05), and the RMS of the COP in the ML direction (*P* < 0.05) during single-leg standing both with and without vision.

**Conclusion:**

The remodeled bicycle pedal training improved the passive JPS and reduced the postural sway in single-leg standing both with and without vision. Therefore, remodeled bicycle pedal training can be considered for inclusion in rehabilitation programs for athletes with FAI to restore the proprioception and balance ability.

## Introduction

Ankle sprain is one of the most common injuries in sports and general activities (Al-Mohrej and Al-Kenani, 2016a). Furthermore, around 20% of patients who suffer intense ankle sprain go on to develop functional ankle instability (FAI) due to lack of functional rehabilitation (Brown et al., 2008; Hopkins et al., 2009; Al-Mohrej and Al-Kenani, 2016b). FAI stems from insufficient muscle strength and impaired neuromuscular control, and is associated with a wide range of problems, including proprioception deficits, delayed muscle reaction, damaged ligaments, and impaired sensation (Wikstrom et al., 2006). Furthermore, FAI can lead to restricted motion, diminished self-reported function, activity disorders, muscle weakness, poor stability, and pain over prolonged periods (Williams et al., 2007; Hertel and Corbett, 2019). As a result, effective FAI treatments are essential in restoring the balance ability and proprioception of athletes.

Static balance is the ability to maintain a support area with minimal sway while the dynamic balance represents that the ability to maintain posture while carrying out tasks or sports skills. Balance ability refers to the control that coordinates the body continuously in relation to the environment (Tracey et al., 2012), which was a complex of static and dynamic balance. A degraded balance capability frequently leads to instability of the ankle joint (Mattacola and Dwyer, 2002; Nam et al., 2018). Consequently, balance exercise training is often prescribed as a treatment for improving postural control, proprioception, balance, and ankle joint stability (Ross and Guskiewicz, 2006; Wortmann and Docherty, 2013; Nam et al., 2018). Jain et al. (2014) showed that a 4-week balance training program successfully reduced ankle instability in patients with FAI. Many studies have performed modified Rhomberg tests to identify the extent to which balance deficits affect individuals with FAI (Freeman et al., 1965; Tropp et al., 1984; Khasnis and Gokula, 2003). In general, the results have shown that the magnitude of the postural sway increases in subjects performing balance tasks with ankle sprain. In addition, individuals with FAI exhibit a greater center of pressure (COP) excursion, COP velocity and time to stabilization in the ML direction than healthy controls (Ross et al., 2009). Consequently, in evaluating potential treatment protocols for individuals with FAI, it is necessary to assess the postural control in the frontal plane in order to ascertain the stability of the subtalar joint (Ross et al., 2009).

Rehabilitation programs such as muscle strengthening, proprioceptive training, balance training and neuromuscular training appear to be effective treatment modes for FAI (Rivera et al., 2017). The current rehabilitation program uses unstable surface such as BOSHU balance trainer, ankle disk, or wobble board, for individuals with ankle instability and show the positive training effects on proprioception or balance (de Brito Silva et al., 2018; Sierra-Guzmán et al., 2018). However, ankle sprain usually occurs in a closed-chain condition under the effects of a sudden high impact force, the aforementioned rehabilitation programs generally focus on only static or low-intensity training. Accordingly, a training program with an unstable surface is more suitable to the daily environment for individuals with FAI. Høiness et al. (2003) investigated the effectiveness of bi-directional (inversion/eversion) bicycle pedal for the rehabilitation of ankle instability. The pedal was designed to tilt 20° in the frontal plane under the effects of loading in order to challenge the ankle stability, joint position sense (JPS), joint movement, and eversion peak torque of the isokinetic muscle strength (Høiness et al., 2003). As such, the bicycle pedal training offered a different intervention from traditional physical training programs, in which the remodeled tri-directional bicycle pedal can move freely and provide unstable pedaling condition. In addition to ordinate ankle plantar flexion, the novel design of remodeled pedal allows extra frontal and transverse plane movement of ankle for inducing peroneus longus activation. In addition, the modified pedal could cope with different bicycle resistance for alternative bodyweight and athlete’s rehabilitation progression until those athletes return to the field. While the traditional physical training programs focused mainly on the static or low intensity training with sagittal plane training. The results showed that participants with recurrent ankle sprains exhibited an improved single-leg stance performance (Høiness et al., 2003) following 6-week bi-directional pedal training.

However, Høiness et al. (2003) considered only bi-directional (inversion/eversion) bicycle pedal training. Furthermore, the effectiveness of the proposed training protocol was evaluated only by static balance tests. Accordingly, the present study performs training using a remodeled tri-directional bicycle pedal capable of moving freely in a specific plane, such as sagittal or frontal plane during the load cycle. In addition to ordinate ankle plantar flexion (PF), the remodeled tri-directional pedal also allows frontal and transverse plane movements of the ankle in order to induce peroneus longus activation. Hence, the purpose of this study was to evaluate the effectiveness of the remodeled tri-directional bicycle pedal training on the passive ankle JPS in dorsal flexion (DF), PF, eversion (EV), and inversion (IV) and the balance ability. This study hypothesized that athletes with FAI demonstrated the improvements on the balance ability and the passive JPS after training.

## Materials and Methods

### Participants

Twenty-six athletes with a self-reported CAIT history were recruited to participate in the study. The CAIT comprises nine items and provides a score in the range of 0 to 30 (Hiller et al., 2006). Generally speaking, any score less than 24 implies severe ankle instability (Donahue et al., 2011). All of the participants performed regular exercise for at least 1 to 2 h every day, 2 to 3 times per week, and engaged in sports involving jump-landing and lateral shuffling tasks, such as basketball, volleyball and soccer. The inclusion criteria of the athletes with FAI were specified as follows: (1) experiencing at least one acute ankle inversion sprain resulting in swelling, pain and protected weight bearing and/or dysfunction of the injured ankle (Monaghan et al., 2006; Brown et al., 2008) in the 6 months prior to the study; (2) having episodes of the ankle giving way in the 3 months prior to the study; (3) suffering from at least one recurrent ankle sprain in the 3 months prior to the study (Brown et al., 2008; Hass et al., 2010); (4) a self-reported CAIT score of less than 24 (Donahue et al., 2011); and (5) negative clinical anterior drawer and talar tilt tests. The participants with FAI were randomly assigned to two groups, namely a training group (AI-T group) and a non-training group (AI-NT group). The athletes in the AI-T group received a 6-week remodeled bicycle pedal training, while those in the AI-NT group received no intervention at all. An additional 14 healthy athletes were also recruited to participate in the study, where the inclusion criteria were specified as: (1) no musculoskeletal disorder in the lower extremities, and (2) a CAIT score equal to or greater than 28 (Monaghan et al., 2006; Miller et al., 2012). The exclusion criteria for all groups were set as follows: (1) having any history of lower extremity fractures or serious orthopedic injury that would affect the performance; (2) having any neurological disorder; (3) suffering from any acute inflammation of the ankle joint; (4) having any history of disorder affecting equilibrium and balance control, and (5) having any head injury at the time of participation. The participants were informed of the experimental procedure before data collection and signed informed consent forms prior to taking part in the study. The study procedures and consent forms were approved by the University Hospital Institutional Review Board (IRB No. B-ER-101-172).

### Equipment

**FIGURE 1:**
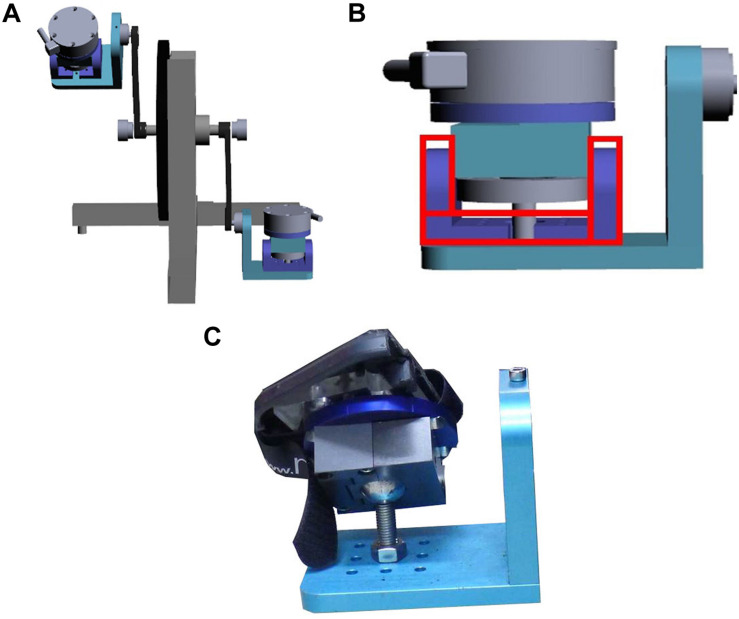
Remodeled bicycle pedal. **(A)** A set of the remodeled pedal. **(B)** The illustration of the U-shape accessory mounted under the remodeled pedal. **(C)** The photograph of the remodeled pedal.

The remodeled bicycle pedal was designed to tilt in range from 0 to 40° sideways during the load cycle in order to mimic the perturbations experienced by an athlete during exercise. A U-shape accessory mounted under the remodeled pedal was to constrain the tilt direction of pedal, while the removal of the U-shape accessory made pedals move freely in three planes (Figure 1). The pedal tilt was passively induced by users during load cycle rather than the pedal provide sudden tilt actively.

One force plate with Bioware software (Kistler Instrumentation Inc., Winterthur, Switzerland) was used to collect force information during single leg standing with/without vision at a sampling rate of 1,000 Hz in order to calculate the COP-related parameters. The Biodex isokinetic dynamometer (Biodex Medical Systems, Shirley, NY, United States) was used to measure the passive JPS.

### Assessments

#### Passive Joint Position Sense Test

**FIGURE 2:**
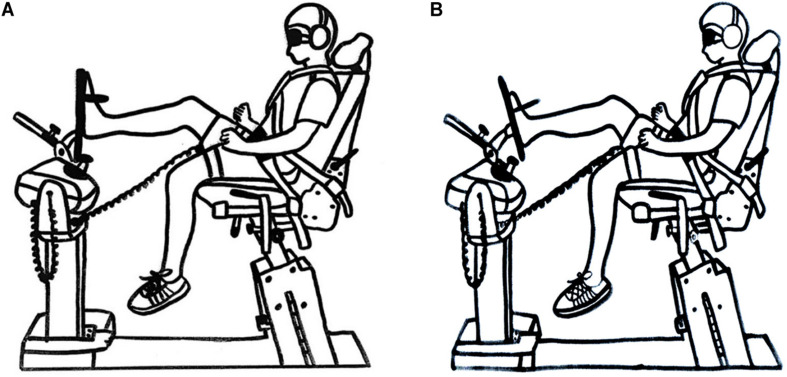
Illustration showing passive joint position sense test arrangement. **(A)** Ankle neutral position. **(B)** Ankle end position.

The passive JPS of the athletes was evaluated using a Biodex isokinetic dynamometer (Biodex Medical Systems Inc., Shirley, NY, United States). Each participant was positioned in the testing chair with the knee of the tested flexed at an angle of 30° and the foot resting on a footplate (Figure 2). The bare foot of the participant was aligned with the axis of the dynamometer and attached to the footplate to reduce cutaneous receptor input. The ankle joint was placed in a neutral position and four angle positions were then randomly tested, namely 15° DF, 15° EV, 15° PF, and 15° IV. The participant’s foot was first passively moved by the investigator to one of the four test positions, randomly determined. The test position was maintained for 10 s, with the participant being instructed to concentrate on the position of the foot. The foot was then brought passively back to the neutral position and then moved passively toward the test position once again with a speed of 0.25° s^–1^ (15 degrees/0.25 degrees per second = 60 seconds). The participant was instructed to push a stop button when he or she thought that the test position had been reached.

#### Single-Leg Standing Tests

Each participant performed single-leg standing tasks in the shod condition with the arms crossed. The participants were asked to raise the sound side foot off the ground and maintain balance for at least 15 s while standing on the affected leg. The tests were performed in both the eyes-closed condition and the eyes-open condition, where in the latter case, the participants were instructed to gaze at a target located at eye level 10 m away.

### Data Reduction

The collected force data were filtered with a 4th order Butterworth low pass filter at 25 Hz. Data reduction was then performed using a custom-made algorithm implemented in MATLAB (R2012a, Mathworks Inc., Natick, MA, United States).

#### Passive Joint Position Sense

For each of the four target positions, the absolute error value of the passive JPS (in degrees) was defined as the difference between the measured angle (i.e., the angle perceived by the participant) and the target position and was noted for further analysis.

#### Displacements, Average Velocity and Sway Ellipse Area of COP

The static force plate measures were analyzed in both the AP and ML directions. For both directions, the excursion was defined as the total length of the COP path, and was approximated by the sum of the distances between consecutive points along the path. The RMS distance of the COP path from the mean COP was evaluated as the standard deviation (SD) of the time series. In addition, the mean velocity (MVELO) was computed as the average velocity of the COP over the total excursion length. (Note that in effect, this normalizes the total excursion to the analysis interval). Finally, the 95% confidence ellipse area was taken as the area of the 95% bivariate confidence ellipse, which was expected to enclose approximately 95% of the points on the COP path. The COP displacements were normalized to the corresponding foot length to eliminate the effects of the foot position and foot configuration factors.

### Training Program

As described above, the 26 participants with FAI were randomly arranged to either a modified bicycle pedal training group (AI-T group) or a non-training group (AI-NT group), with 13 members in each group. Participants in the AI-T group were subsequently excluded from the study if they failed to complete at least 80% of the training program, while participants in the AI-NT group were excluded if they did not perform regular exercise for at least 1 h a day, 2 days a week (as evidenced in a training record and daily log, respectively).

For the AI-T group, the heart rate during pedaling was evaluated by the “telemetry heart rate,” which was attached around the subject’s chest. Each training session commenced with a 5-min warm-up period at 50% target heart rate. Specifically, the bicycle resistance was set to Level 1 (low resistance) for 1 min and was then increased to 0.077 body weight of the corresponding participant (Tossavainen et al., 1996) for a further 3 min. The resistance was then returned to Level 1 and the protocol was repeated for 40 min per session with 3 sessions a week.

The intensity of the training was set in accordance with the heart rate reserve method, which takes the difference of the maximum heart rate and resting heart rate into consideration (Høiness et al., 2003). Each training session commenced with a pedaling rate ranged from 50 to 70 rpm in order to reach the target exercise intensity (Høiness et al., 2003). The intensity of the training session was measured using the rated perceived exertion (RPE) scale (Borg, 1962; Lollgen et al., 1975), which runs from 6 to 20, where a higher value indicates a greater level of exertion. The participants were expected to achieve a minimal RPE score of 13, representing as somewhat hard during training. The participants were additionally requested to report their subjective pain using the visual analog scale (VAS) (McCormack et al., 1988; Bijur et al., 2001).

The main purpose of simulating ankle instability in frontal plane during training session was to facilitate specific muscles to maintain ankle stability. As such, the U-shape accessory was used to constrain the pedal to tilt in frontal plane (inversion/eversion) in the first 3 weeks training session while the U-shape accessory was removed to tilt the pedal in three planes for the remaining 3 weeks.

### Procedures

Investigators explained the study purposes and the procedures to all participants and participants signed an informed consent form before assessments and training sessions. Firstly, participants were requested to perform passive JPS tests in four ankle positions for five repetitions each. Then, participants carried out single leg standing with/without vision with each condition performing five repetitions. After completing all baseline measurements, participants in AI-T group received 6 weeks training with remodeled pedal while participants in the AI-NT and the H-group received no training. All assessments were measured again for AI-T and AI-NT groups after 6 weeks, whereas, H-group received baseline measurement only.

### Statistical Analysis

SPSS version 17.0 (SPSS Inc., Chicago, IL, United States) was used for all the statistical analyses. The potential intervention effects were tested by the analysis of covariance (ANCOVA). In addition, one-way ANOVA with Tukey’s *post hoc* test or the Kruskal Wallis test was used to test for differences among the AI-T group, AI-NT group and H-group in the group demography and the post-training test condition depends on the data normality. The dependent variables were absolute JPS errors and the COP-related parameters during single leg standing with or without vision. An alpha level of *P* < 0.05 was considered to be statistically significant.

## Results

### Demographics

No significances were found among three groups in age (AI-T: 22.66 ± 2.47 yr, AI-NT: 22.86 ± 1.78 yr; H-group: 21.37 ± 0.75 yr, *p* = 0.068), height (AI-T: 168.75 ± 6.73 cm, AI-NT: 166.90 ± 7.95 cm; H-group: 170.84 ± 7.53 cm, *p* = 0.373), and body weight (AI-T: 67.88 ± 13.68 kg, AI-NT: 62.22 ± 8.83 kg; H-group: 65.14 ± 10.90 kg, *p* = 0.418). Regarding to the regular exercise volume per week, no significance difference was found among three groups (AI-T: 11.62 ± 4.93 h, AI-NT: 11.00 ± 4.34 h, and H-group: 9.32 ± 3.11 h, *p* = 0.242). The comparisons of baseline measurements in passive JPS, COP-related parameters during single leg standing with/without vision among groups were presented in Supplementary Material.

### Passive Joint Position Senses

The AI-T group demonstrated significantly decreased absolute ankle JPS error in all directions compared to the AI-NT group after 6 week training (*P* < 0.05) (Table 1).

**TABLE 1:** ANCOVA analysis of absolute passive JPS errors for training group and non-training group after 6 weeks with baseline measurement as covariate.

Outcome variables		Groups		ANCOVA (pre-test as covariate)	
		AI-T (Mean ± SD)	AI-NT (Mean ± SD)	F statistics	*P-values*
Dorsi-flexion	Baseline	1.67 ± 0.11	1.39 ± 0.47	–	–
	Follow-up	0.92 ± 0.25	1.98 ± 0.75	29.33	<0.001
Plantar-flexion	Baseline	2.67 ± 0.52	3.45 ± 0.73	–	–
	Follow-up	1.09 ± 0.37	2.75 ± 0.33	98.77	<0.001
Inversion	Baseline	2.37 ± 0.38	2.79 ± 1.02	–	–
	Follow-up	0.87 ± 0.35	2.6 ± 0.33	15.34	0.001
Eversion	Baseline	3.67 ± 0.54	3.05 ± 1.41	–	–
	Follow-up	1.07 ± 0.42	1.78 ± 0.57	162.35	<0.001

After a 6-week training, a significant difference was observed among the three groups (AI-T, AI-NT, and H-group) with regard to the absolute JPS errors in DF (*p* = 0.003), PF (*p* < 0.001), IV (*p* < 0.001), and EV (*p* < 0.001) (Table 2).

**TABLE 2:** One-way ANOVA and post hoc test results for absolute passive JPS errors of three groups after 6-week training.

	Groups			P-values	Post hoc comparisons
	AI-T (Mean ± SD)	AI-NT (Mean ± SD)	Healthy (Mean ± SD)		
Dorsi-flexion	0.92 ± 0.25	1.98 ± 0.75	1.25 ± 0.65	0.003	a
Plantar-flexion	1.09 ± 0.37	2.75 ± 0.33	2.98 ± 0.79	< 0.001	a; b
Inversion	1.07 ± 0.35	2.60 ± 0.33	2.39 ± 0.83	< 0.001	a; b
Eversion	0.87 ± 0.42	1.78 ± 0.57	2.62 ± 1.00	<0.001	a; b

*^a^Represents the significant differences exist between AI-T and AI-NT. ^b^Represents the significant differences exist between AI-T and Healthy.*

According to the *post hoc* tests, the absolute JPS error in DF, PF, IV, and EV (*p* < 0.05) were significantly smaller in the AI-T group than in the AI-NT group after 6-week training. In addition, the absolute JPS error in PF, IV, and EV (*p* < 0.05) were significantly lower in the AI-T group than in the H-group (Table 2). However, there were no significant differences existed between the AI-NT group and the H-group.

### Single-Leg Standing Tests

#### Single-Leg Standing With Eyes Opened

The sway excursion in ML, sway velocity in ML, RMS in ML, and 95% ellipse sway area (*P* < 0.05) were found to be lower in the AI-T group than in the AI-NT group after training (Table 3). However, there were no significant differences observed in the postural sway related parameters in AP direction between AI-T group and AI-NT group (Table 3).

**TABLE 3:** ANCOVA analysis of postural sway variables for training group and non-training group during single leg stance with vision after 6 weeks with baseline measurement as covariate.

Outcome variables		Groups		ANCOVA (pre-test as covariate)	
		AI-T (Mean ± SD)	AI-NT (Mean ± SD)		
				*F* statistics	*P-values*
AP excursion (cm)	Baseline	2.33 ± 0.47	2.47 ± 0.60		
	Follow-up	2.31 ± 0.46	2.58 ± 0.73	2.32	0.132
ML excursion (cm)	Baseline	5.35 ± 1.74	5.56 ± 1.22		
	Follow-up	5.11 ± 1.38	6.09 ± 1.84	8.65	0.004
AP velocity (cm/s)	Baseline	0.12 ± 0.02	0.12 ± 0.03		
	Follow-up	0.12 ± 0.02	0.13 ± 0.04	2.32	0.132
ML velocity (cm/s)	Baseline	0.27 ± 0.09	0.28 ± 0.06		
	Follow-up	0.26 ± 0.07	0.30 ± 0.09	8.65	0.004
AP RMS (cm)	Baseline	0.02 ± 0.004	0.03 ± 0.003		
	Follow-up	0.02 ± 0.01	0.30 ± 0.07	0.59	0.445
ML RMS (cm)	Baseline	0.09 ± 0.02	0.09 ± 0.02		
	Follow-up	0.09 ± 0.02	0.10 ± 0.03	5.50	0.022
95% ellipse area (cm^2^)	Baseline	0.03 ± 0.01	0.03 ± 0.01		
	Follow-up	0.03 ± 0.01	0.03 ± 0.02	5.74	0.019

AI-T group demonstrated significant smaller sway excursion in ML and sway velocity in ML than that in the AI-NT group (*P* < 0.05), however, there were no significant differences between AI-T group and the H-group. After 6 weeks, the sway excursion in ML, sway velocity, RMS in ML, and 95% ellipse sway area in the AI-NT group were significantly greater than that in the H-group (*P* < 0.05) (Table 4). Nevertheless, there were no significant differences existed among groups in postural sway related parameters in AP direction (Table 4).

**TABLE 4:** One-way ANOVA and post hoc test results for postural sway variables during single leg stance with vision of three groups after 6 weeks.

	Group			*P*-values	*Post hoc* comparisons
	AI-T (Mean ± SD)	AI-NT (Mean ± SD)	Healthy (Mean ± SD)		
AP excursion (cm)	2.31 ± 0.46	2.58 ± 0.73	2.27 ± 0.45	0.202	–
ML excursion (cm)	5.11 ± 1.38	6.09 ± 1.84	4.77 ± 1.13	0.004	a; b
AP velocity (cm/s)	0.12 ± 0.02	0.13 ± 0.04	0.11 ± 0.02	0.202	–
ML velocity (cm/s)	0.26 ± 0.07	0.30 ± 0.09	0.24 ± 0.06	0.004	a; b
AP RMS (cm)	0.02 ± 0.01	0.03 ± 0.01	0.02 ± 0.004	0.158	–
ML RMS (cm)	0.09 ± 0.02	0.10 ± 0.03	0.08 ± 0.02	0.012	b
95% ellipse area (cm^2^)	0.03 ± 0.01	0.03 ± 0.02	0.02 ± 0.01	0.011	b

*^a^Represents the significant differences exist between AI-T and AI-NT. ^b^Represents the significant differences exist between AI-NT and Healthy.*

#### Single-Leg Standing Test With Eyes Closed

After training, AI-T group demonstrated smaller sway excursion both in AP and ML, sway velocity in ML, and RMS in both AP and ML (*P* < 0.05) during single (Table 5). However, there were no significant differences observed in the postural sway related parameters in AP direction between AI-T group and AI-NT group (Table 5).

**TABLE 5:** ANCOVA analysis of postural sway variables for training group and non-training group during single leg stance without vision after 6 weeks with baseline measurement as covariate.

Outcome variables		Groups		ANCOVA (pre-test as covariate)	
		AI-T (Mean ± SD)	AI-NT (Mean ± SD)	*F* statistics	*P-values*
AP excursion (cm)	Baseline	2.70 ± 0.77	2.52 ± 0.57		
	Follow-up	2.27 ± 0.45	2.49 ± 0.75	6.03	0.017
ML excursion (cm)	Baseline	6.73 ± 2.35	5.56 ± 1.47		
	Follow-up	5.02 ± 1.41	5.48 ± 1.96	11.28	0.001
AP velocity (cm/s)	Baseline	0.26 ± 0.09	0.25 ± 0.06		
	Follow-up	0.23 ± 0.05	0.25 ± 0.08	3.13	0.081
ML velocity (cm/s)	Baseline	0.65 ± 0.24	0.56 ± 0.15		
	Follow-up	0.48 ± 0.15	0.55 ± 0.20	10.19	0.002
AP RMS (cm)	Baseline	0.04 ± 0.01	0.05 ± 0.01		
	Follow-up	0.04 ± 0.01	0.05 ± 0.01	9.71	0.003
ML RMS (cm)	Baseline	0.16 ± 0.05	0.12 ± 0.03		
	Follow-up	0.11 ± 0.03	0.13 ± 0.03	27.46	< 0.001
95% ellipse area (cm^2^)	Baseline	0.17 ± 0.04	0.07 ± 0.02		
	Follow-up	0.05 ± 0.02	0.07 ± 0.03	0.969	0.329

AI-T group demonstrated significant smaller sway excursion in ML and sway velocity in ML than that in the AI-NT group (*P* < 0.05), however, there were no significant differences between AI-T group and the H-group. After 6 weeks, the sway excursion in ML, sway velocity, RMS in ML, and 95% ellipse sway area in the AI-NT group were significantly greater than that in the H-group (Table 6). Nevertheless, there were no significant differences existed among groups in postural sway related parameters in AP direction (Table 6).

**TABLE 6:** One-way ANOVA and post hoc test results for postural sway variables during single leg stance without vision of three groups after 6 weeks.

	Group			*P*-values	*Post hoc* comparisons
	AI-T (Mean ± SD)	AI-NT (Mean ± SD)	Healthy (Mean ± SD)		
AP excursion (cm)	2.27 ± 0.45	2.49 ± 0.75	2.38 ± 0.42	0.528	–
ML excursion (cm)	5.02 ± 1.41	5.48 ± 1.96	5.22 ± 1.21	0.899	–
AP velocity (cm/s)	0.23 ± 0.05	0.25 ± 0.08	0.24 ± 0.04	0.528	–
ML velocity (cm/s)	0.48 ± 0.15	0.55 ± 0.20	0.52 ± 0.12	0.499	–
AP RMS (cm)	0.04 ± 0.01	0.05 ± 0.01	0.04 ± 0.01	0.019	a
ML RMS (cm)	0.11 ± 0.03	0.13 ± 0.03	0.11 ± 0.03	0.016	a; b
95% ellipse area (cm^2^)	0.05 ± 0.02	0.07 ± 0.03	0.06 ± 0.03	0.002	a

*^a^Represents the significant differences exist between AI-T and AI-NT. ^b^Represents the significant differences exist between AI-NT and Healthy.*

## Discussion

This study investigated the effects of remodeled pedal training for athletes with ankle instability, and compared to those received no training or the healthy controls. The exercise volume and training experiences might be potential factors to influence the outcomes. However, the exercise volume did not have significant differences among three groups. In addition, the training experiences of participants recruited in this study including basketball, volleyball, gymnastics, handball, traditional folk dance, and high jump that are diverse in training experiences. With randomized control design, both exercise volume and training experiences should have very little effect on our results.

With regard to the pedal design, the regular bicycle pedal produces dorsiflexion-PF during cycling whereas the remodeled pedal produces not only the movement in the sagittal plane, but also the unexpected movement in the frontal plane. The active ankle range of motion in athletes with chronic ankle sprain is 10.56 ± 3.67° in for dorsiflexion, 40.61 ± 4.91° in PF, 30.92 ± 3.70° in inversion, and 17.61 ± 1.35° in eversion (Alghadir et al., 2020). The maximal tilt angle of the remodeled pedal was 40°. Thus, the remodeled pedal can simulate regular bicycle movement and the ankle range of motion was close to athletes with chronic ankle sprain.

Briefly summarizing the results of this study, the ANCOVA controlling for baseline measurements, indicated that participants in the AI-T group had significantly better performance in passive JPS and postural sway than participants in the AI-NT group. These results supported our hypotheses and this might be because of the fact that the remodeled pedal is capable of moving freely to simulate ankle instability during the load cycle, hence, participants in the AI-T group have to manipulate the ankle position and maintain ankle stability deliberately and further improved the passive JPS.

### Passive Joint Position Sense

Joint position sense is a specialized sensory modality which governs the ability of a joint to determine its position in space, detect movement, and sense resistance acting on it (Riemann and Lephart, 2002). Docherty et al. (1998) examined the effects of ankle strengthening exercises on JPS in subjects with FAI and reported that the strength training led to increased gamma-efferent activity. In addition, the authors suggested that the spindle was more sensitive to instantaneous stretching; resulting in an improved acuity in JPS (Docherty et al., 1998). It has been reported that the primary sensory endings of muscle spindles detect the velocity of stretching as well as the relative amount of stretch (Pearson, 2000). The tri-directional pedal employed in the present study was designed to create an unstable plane by tilting suddenly as the participants unconsciously manipulated their ankle position. It was speculated that the resulting instantaneous stretch would enhance the acuity of JPS and thus serve to re-educate the proprioceptive system by improving the mechanoreceptor function and restoring normal neuromuscular coordination. This may further explain why the outcomes of this 6-week remodeled pedal training were superior to those of healthy control group.

In the current study, the AI-T group demonstrated significantly better passive JPS of the ankle in all directions after a 6-week remodeled bicycle pedal training. In addition, the absolute passive JPS errors in PF, IV, and EV were lower in the AI-T group than in the AI-NT group in the post-training condition and the H-group. These findings are consistent with most previous studies. For example, individuals with FAI who underwent 12-week rehabilitation with a biomechanical ankle platform system (BAPS) showed a reduced absolute JPS error in both active and passive reposition tests (Lee and Lin, 2008). Similarly, several studies have shown that wobble board and tilting board coordination training for individuals with FAI leads to a significant improvement of proprioception in ankle inversion (Bernier and Perrin, 1998; Wright et al., 2020). In addition, a 6-week multi-station proprioceptive training program resulted in a positive effect on the JPS in individuals with FAI in both 15° PF and 30° PF (Eils and Rosenbaum, 2001). Notably, the AI-NT group in the present study showed no significant change in passive JPS between pre-test and post-test. Hence, the possibility of spontaneous recovery in the AI-T group can be effectively ruled out.

The findings of the studies above present that training with a BAPS system, wobble board, tilting board improves the proprioception in subjects with FAI. Accordingly, the remodeled pedal employed in the present study was designed specifically to reproduce the same joint perturbation as those in the above-mentioned studies. In particular, the pedal was designed to facilitate maximal stimulation of the ankle joint, muscle and mechanoreceptors. Thus, during training, the athletes were required to deliberately maintain their ankle position and preserve pedal stability while pedaling. It is speculated that the enhanced ankle proprioception observed in the AI-T group may result from the reconstruction and integration of the neuromuscular control of the ankle joint during pedal training. Neuromuscular function relies on mechanoreceptors, efferent and afferent nerve fibers, the central nervous system, and the strength of the ankle stabilizing muscles (Høiness et al., 2003). Furthermore, muscle strengthening may create compensatory pathways for receiving more afferent information (Lee and Lin, 2008). Thus, muscle training on a multiaxial support surface needs particular muscle-stabilization mechanisms and a faster stabilization response from the ankle mechanoreceptors.

### Single-Leg Standing

#### Eyes-Open Condition

Postural control is an outcome of the integration of visual, somatosensory, and vestibular information (Schmidt, 1975). Many studies have shown that vision helps maintain body orientation in space (Sheldon, 1963). Once visual feedback is removed, a greater dependence on mechanoreceptor and vestibular information occurs. The difference between unstable and stable ankles relies on the ability of the mechanoreceptors to control postural sway. Thus, if the mechanoreceptors do not sufficiently sense the changes in tension occurring within the joint, and hence fail to provide the proprioceptive information correctly, insufficient control movement may be applied; resulting in joint instability.

Many researchers have shown that postural sway increases when balancing on unstable ankles (Ross and Guskiewicz, 2004; Michell et al., 2006). In the present study, during single-leg standing with the vision, the AI-T group demonstrated significantly lower sway excursion in ML, sway velocity in ML, RMS in ML, and 95% ellipse sway area than in the AI-NT group. On the other hand, the H-group demonstrated smaller postural sway excursion, velocity, RMS, and 95% ellipse sway area than in the AI-T group. However, there were no significant differences between the AI-T group and the H-group after 6 weeks training. These findings are consistent with many previous studies. For example, Ross et al. (2009) showed that with vision, unstable ankles exhibit a greater ML mean COP velocity, ML COP excursion and ML mean COP excursion than stable ankles. Similarly, Mitchell et al. (2008) demonstrated that in the eyes-open condition, individuals with FAI show a greater AP sway than healthy individuals. In the AI-NT group, the musculature controlling movements cannot function in the same way as those in the H-group due to a decreased JPS or reduced proprioception in the mechanoreceptors monitoring the postural sway (Mitchell et al., 2008).

#### Eyes-Closed Condition

Mitchell et al. (2008) reported a greater ML postural sway in individuals with FAI than in healthy subjects when performing single-leg standing without vision. Hence, ML postural sway is an important parameter for detecting the effect of training for participants with FAI. The present results have shown that pedal training reduces the excursion of postural sway in both AP and ML, velocity of postural sway in ML, and the RMS of postural sway in both AP and ML directions compared to the AI-NT group, in addition, these parameters did not have significant differences between AI-T group and the H-group after training. These findings are consistent with those of Høiness et al. (2003) who showed that following high-intensity pedal training with a bi-directional pedal, the subjects increased their recovery speed to maintain balance during single-leg stance tasks.

The AP and ML parameters of postural sway reduced following 10-week BAPS training (Hoffman and Payne, 1995). Similarly, subjects with FAI showed a lower mean radius of the COP during single-leg standing with and without vision after 12-week BAPS training (Lee and Lin, 2008). Furthermore, 6-week multi-direction proprioception exercise improved the AP postural sway (SD and maximal sway range) and total sway distance of individuals with ankle instability (Eils and Rosenbaum, 2001). During unstable plane training, subjects must manipulate their ankle and maintain balance when the surface suddenly drops, which requires prompt and efficient muscle co-activation. The mechanisms required for such rapid stabilization originate from neuromuscular control, and depend on adequate joint proprioception and muscle strength (Høiness et al., 2003; Lee and Lin, 2008).

In the present study, the passive JPS of the AI-T group improved following 6-week pedal training. It is thus speculated that the training effect enabled the athletes to maintain better postural control during single-leg stance as a result of improved joint stabilization due to an enhanced proprioception and muscle strength of the ankle joint.

Postural control demands afferent information from the visual, vestibular and somatosensory systems, and provides an efferent response that includes both muscle contraction and reflex. When visual input is blocked, postural control depends exclusively on the vestibular and somatosensory systems. The present study excluded athletes with an impaired vestibular system. Consequently, it can be inferred that an improved proprioception of the mechanoreceptors around the ankle joint was observed after pedal training. Moreover, proprioception provides the motor programming required by neuromuscular control to perform precise movements and also facilitates dynamic joint stability and postural control stability (Lephart et al., 1997). Ankle sprain results in JPS decrements, which lead in turn to deficits in neuromuscular control. Furthermore, the muscle spindle is more sensitive under instantaneous stretching, resulting in greater acuity of the JPS (Docherty et al., 1998). Consequently, the pedal training performed in the present study may affect the muscle response (the efferent component) and hence further affect the passive JPS and postural sway.

### Limitations

In the present study, the number of male participants was twice that of the female participants. Thus, the variability among the athletes observed in the various tests and conditions may be influenced by differences in the gender and physical activity within the same group. For example, the inversion-eversion angles of female athletes are generally greater than those of male athletes (Ericksen and Gribble, 2012). So, further investigation is essential to address this limitation. In addition, the foot position of the athletes during pedal training was fixed by a strap. However, it is likely that the foot position changed slightly during pedaling, and had a subsequent impact on the training effects. Accordingly, in a future study, the foot position should be fixed more securely to ensure appropriate foot-leg alignment and pedal stability. Additionally, this study did not include the traditional cycling training group for comparison and thus cannot evaluate whether this remodeled pedal training is superior to traditional cycling training.

## Conclusion

A remodeled bicycle pedal has been designed to imitate an unstable ankle condition. In performing the remodeled bicycle pedal training, the participants were required to deliberately manipulate the ankle position and maintain ankle stability. This leads to stimulation of the ankle joint, muscle and mechanoreceptors, and therefore improves the acuity of JPS in all directions (dorsiflexion, plantarflexion, inversion, and eversion). Consequently, the training program improves the proprioception at the ankle joint, and hence contributes to better postural balance during single leg stance with or without vision.

The 6-week remodeled pedal training program reduced the postural sway during single-leg standing in both the eyes-open and eyes-closed conditions. As such, it appears to merit serious consideration for inclusion in the rehabilitation programs of individuals with FAI to restore the proprioception and the balance ability.

## Data Availability Statement

All datasets presented in this study are included in the article/Supplementary Material.

## Ethics Statement

The studies involving human participants were reviewed and approved by Institutional Review Board, National Cheng Kung University Hospital. The patients/participants provided their written informed consent to participate in this study. Written informed consent was obtained from the individual(s) for the publication of any potentially identifiable images or data included in this article.

## Author Contributions

Y-SC, C-FL, and F-CS: conceptualization. Y-SC, L-CK, F-CS, H-WW, and C-FL: methodology. Y-SC, H-WW, Y-LY, and C-FL: software and formal analysis. Y-SC, H-WW, and C-FL: investigation. MA, Y-SC, C-FL: writing – original draft preparation. MA, Y-SC, Y-LY, L-CK, F-CS, and C-FL: writing – review and editing. All authors contributed to the article and approved the submitted version.

## Conflict of Interest

The authors declare that the research was conducted in the absence of any commercial or financial relationships that could be construed as a potential conflict of interest.
