# Supplementary material for: Effect of Novel Remodeled Bicycle Pedal Training on Balance Performance in Athletes With Functional Ankle Instability
Source: Front Bioeng Biotechnol. 2020 Oct 22;8:600187. doi: 10.3389/fbioe.2020.600187 (PMC7642596; doi:10.3389/fbioe.2020.600187)
Supplement: Supplementary file 1 [file Table_1.DOCX]

|  | Groups | | | P-values | Post-hoc  comparisons |
| --- | --- | --- | --- | --- | --- |
|  | AI-T  (Mean ± SD) | AI-NT  (Mean ± SD) | Healthy  (Mean ± SD) |  |  |
| Dorsi flexion | 1.67 ± 0.11 | 1.39 ± 0.47 | 1.25±0.65 | 0.082 | - |
| Plantar flexion | 2.67 ± 0.52 | 3.45 ± 0.73 | 2.98±0.79 | 0.023 | a |
| Inversion | 2.37 ± 0.38 | 2.79 ± 1.02 | 2.39±0.83 | 0.319 | b |
| Eversion | 3.67 ± 0.54 | 3.05 ± 1.41 | 2.62±1.00 | 0.048 | - |

Supplementary Data

Table 1. One-way ANOVA and post hoc test results for absolute passive JPS errors of three groups at the baseline measurements.

Note: a represents the significant differences exist between AI-T and AI-NT; b represents the significant differences exist between AI-T and Healthy.

|  | Group | | | P-values | Post-hoc  comparisons |
| --- | --- | --- | --- | --- | --- |
|  | AI-T  (Mean ± SD) | AI-NT  (Mean ± SD) | Healthy  (Mean ± SD) |  |  |
| AP excursion (cm) | 2.33 ± 0.47 | 2.47 ± 0.60 | 2.27 ± 0.45 | 0.191 | - |
| ML excursion (cm) | 5.35 ± 1.74 | 5.56 ± 1.22 | 4.77 ± 1.13 | 0.054 | - |
| AP velocity (cm/s) | 0.12 ± 0.02 | 0.12 ± 0.03 | 0.11 ± 0.02 | 0.191 | - |
| ML velocity (cm/s) | 0.27 ± 0.09 | 0.28 ± 0.06 | 0.24 ± 0.06 | 0.054 | - |
| AP RMS (cm) | 0.02 ± 0.004 | 0.03 ± 0.003 | 0.02 ± 0.004 | 0.055 | - |
| ML RMS (cm) | 0.09 ±0.02 | 0.09 ± 0.02 | 0.08 ± 0.02 | 0.049 | b; c |
| 95% ellipse area (cm^2^) | 0.03 ± 0.01 | 0.03 ± 0.01 | 0.02 ± 0.01 | 0.016 | b; c |

Table 2. One-way ANOVA and post hoc test results for postural sway variables during single leg stance with vision of three groups at the baseline measurements.

Note: b represents the significant differences exist between AI-T and Healthy; c represents the significant differences exist between AI-NT and Healthy.

|  | Group | | | P-values | Post-hoc  comparisons |
| --- | --- | --- | --- | --- | --- |
|  | AI-T  (Mean ± SD) | AI-NT  (Mean ± SD) | Healthy  (Mean ± SD) |  |  |
| AP excursion (cm) | 2.70 ± 0.77 | 2.52 ± 0.57 | 2.38 ± 0.42 | 0.087 | - |
| ML excursion (cm) | 6.73 ± 2.35 | 5.56 ± 1.47 | 5.22 ± 1.21 | 0.001 | a; c |
| AP velocity (cm/s) | 0.26 ± 0.09 | 0.25 ± 0.06 | 0.24 ± 0.04 | 0.463 | - |
| ML velocity (cm/s) | 0.65 ± 0.24 | 0.56 ± 0.15 | 0.52 ± 0.12 | 0.011 | a; c |
| AP RMS (cm) | 0.04 ± 0.01 | 0.05 ± 0.01 | 0.04 ± 0.01 | 0.673 | - |
| ML RMS (cm) | 0.16 ± 0.05 | 0.12 ± 0.03 | 0.11 ± 0.03 | <0.001 | a; c |
| 95% ellipse area (cm^2^) | 0.17 ± 0.04 | 0.07 ±0.02 | 0.06 ± 0.03 | <0.001 | a; c |

Table 3. One-way ANOVA and post hoc test results for postural sway variables during single leg stance without vision of three groups at the baseline measurements

Note: a represents the significant differences exist between AI-T and AI-NT; c represents the significant differences exist between AI-NT and Healthy.
